# Combined clinical features and MRI parameters for the prediction of VEGFR2 in hepatocellular carcinoma patients

**DOI:** 10.3389/fonc.2022.961530

**Published:** 2022-10-13

**Authors:** Laizhu Zhang, Chunxiao Cheng, Binghua Li, Jun Chen, Jin Peng, Yajuan Cao, Yang Yue, Xiaoli Mai, Decai Yu

**Affiliations:** ^1^ Hepatobiliary and Pancreatic Center & Liver Transplantation Center, the Affiliated Drum Tower Hospital, Medical School of Nanjing University, Nanjing, China; ^2^ Nanjing Drum Tower Hospital Clinical College of Nanjing University of Chinese Medicine, Nanjing, China; ^3^ Department of Pathology, the Affiliated Drum Tower Hospital, Medical School of Nanjing University, Nanjing, China; ^4^ Department of Radiology, the Affiliated Drum Tower Hospital, Medical School of Nanjing University, Nanjing, China

**Keywords:** hepatocellular carcinoma, LI-RADS, VEGFR2, nomogram, targeted therapy

## Abstract

**Purpose:**

To develop a prediction model for estimating the expression of vascular endothelial growth factor receptor 2 (VEGFR2) in hepatocellular carcinoma (HCC) patients using clinical features and the contrast-enhanced MRI Liver Imaging Reporting and Data System (LI-RADS).

**Methods:**

A total of 206 HCC patients were subjected to preoperative contrast-enhanced MRI, radical resection, and VEGFR2 immunohistochemistry labeling. The intensity of VEGFR2 expression was used to split patients into either the positive group or the negative group. For continuous data, the Mann-Whitney U test was employed, and for categorical variables, the χ2 test was utilized.

**Results:**

VEGFR2-positivity was identified in 41.7% (86/206) of the patients. VEGFR2-positive HCCs were confirmed by higher serum alpha-fetoprotein (AFP) levels, larger tumor dimensions (either on MRI or upon final pathology), and a higher LI-RADS score (all p < 0.001). LI-RADS scores and AFP levels were independent predictors for high VEGFR2 expression. These two parameters were used to establish a VEGFR2-positive risk nomogram, which was validated to possess both good discrimination and calibration. The area under the curve was 0.830 (sensitivity 83.6%, specificity 72.5%) and the mean absolute error was 0.021. The threshold probabilities ranged between 0.07 and 0.95, and usage of the model contributed net benefits.

**Conclusion:**

A nomogram including clinical features and contrast-enhanced MRI parameters was developed and was demonstrably effective at predicting VEGFR2 expression in HCC patients.

## Introduction

Liver cancer was the sixth most common cancer and the fourth leading cause of cancer death worldwide in 2018 (1). Hepatocellular carcinoma (HCC) was the most common kind of liver cancer, accounting for the vast majority of diagnoses and fatalities (2). Because the symptoms of HCC are relatively insidious, many patients have lost the opportunity for surgery when they are diagnosed. For advanced HCC, the treatment options are limited. Targeted therapy is one of the main choices for advanced HCC, with many associated molecular targeted drugs entering the clinic (3).

Vascular endothelial growth factor receptor 2 (VEGFR2) mRNA levels and expression are upregulated in HCC (4). The proliferation, migration, survival, and permeability of vascular endothelial cells are influenced by VEGF/VEGFR2 signaling cascades (5, 6), and these factors can modulate the efficacy of sorafenib therapy. Prior to sorafenib therapy, a lack of VEGFR2 expression in resected tumor tissues is linked to worse overall survival (7). Therefore, anti-angiogenic drugs that block the VEGFR/VEGFR2 pathway provide a new target for the clinical therapy of HCC, and many studies have demonstrated their validity and safety in advanced HCC (8, 9). VEGFR2 is the only target of apatinib and one of the main targets of other targeted drugs, including sorafenib and lenvetinib (10–12). As a result, VEGFR2 expression has important clinical significance due to its susceptibility to targeted therapy in HCC patients (7).

Preoperative examinations like contrast-enhanced MRI can dynamically display the blood supply of lesions and provide perfusion parameters. Furthermore, when combined with the LI-RADS score 2018 (13), these data can provide an assessment of the possibility of malignancy in liver nodules in the background of cirrhosis (14, 15). It has been demonstrated that the signal intensity in the arterial phase as well as the intensity heterogeneity are both positively correlated with VEGF expression in HCC (16). The perfusion markers of blood flow, blood volume in the artery phase, and the hepatic perfusion index were found to be higher with the increasing expression of VEGFR2, as determined by immunostaining (17, 18).

If we can effectively predict the expression of VEGFR2 in patients before undergoing pathological examination, more precise and individualized treatment plans can be made. Therefore, our objective was to find significant predictors for high VEGFR2 expression and to further develop a risk model for its prediction.

## Materials and methods

### Patients

**Figure 1:**
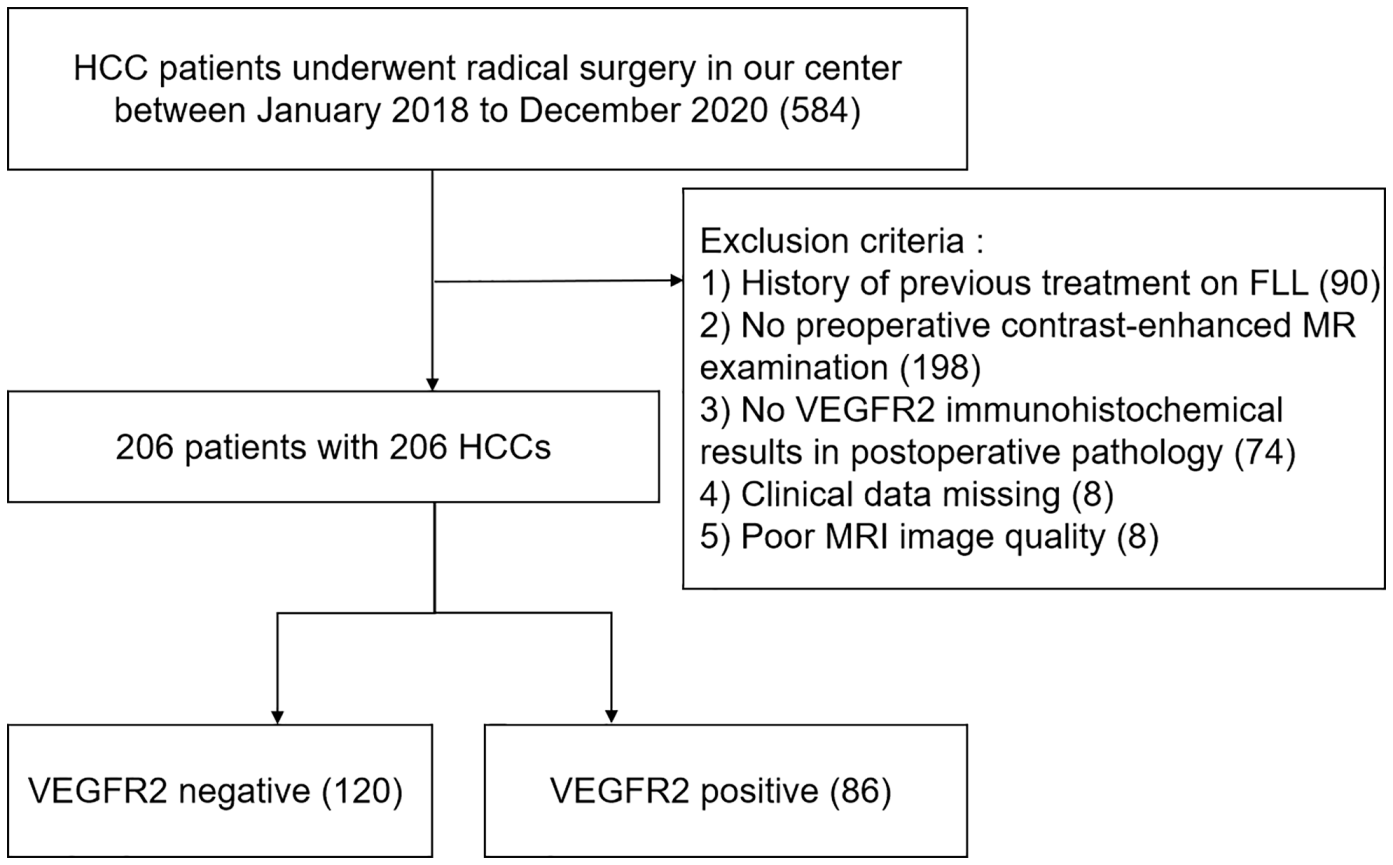
Flowchart of patient selection. HCC, hepatocellular carcinoma; MRI, contrast-enhanced magnetic resonance imaging; FLL, focal liver lesion; VEGFR2, vascular endothelial growth factor receptor 2.

Data was chosen from individuals who had either liver resections or transplants at our center from January 2018 to December 2020. The inclusion criteria were as follows: (1) MRI examination within the 2 weeks prior to surgery; (2) MRI images could clearly recognize the tumor; (3) the presence of primary HCC with no preoperative treatment, such as ablation, chemotherapy, immunotherapy or transarterial chemoembolization; (4) postoperative pathology confirmed the presence of HCC; (5) the clinical data was complete. A total of 206 participants were enrolled in our study (**Figure 1**). Twenty-six patients could not be contacted after radical resection in the follow-up. Thirty-five underwent liver transplants. The hospital review board gave its permission and all of the participants signed their written informed consent.

### MRI examination

All MRI examinations were completed within two weeks prior to radical surgery. Patients were scanned in the supine position using a 3.0-T whole-body MRI scanner (uMR770, United Imaging, China) with a sixteen-channel phased-array coil centered over the belly. Before the MRI, all patients were required to fast for a minimum of 8 hours. A T1-weighted volume interpolated breath-hold gradient recall echo sequence was used to provide an unenhanced scan, dynamic contrast-enhanced phase, and hepatobiliary phase (HBP). The dynamic contrast-enhanced scan early arterial (EAP) and late arterial phases (LAP) were used to ensure that we could observe the enhancement of the tumor. A dosage of 0.025 mmol/kg Gd-EOB-DTPA (Primovist; Bayer HealthCare, Berlin, Germany) was administered at a rate of 2.0 mL/s, and then followed by a 30-mL saline flush at the same rate. Using bolus triggering, a dual arterial phase sequence was started 15-25 seconds after the contrast media arrived at the distal thoracic aorta, while the portal venous phase (PVP), delay phase (DP), and HBP were acquired at 1, 3, and 15 minutes after Gd-EOB-DTPA administration, respectively. The HBP images were used to assist in the diagnosis and demarcation of the tumor location.

### Image analysis and LI-RADS interpretation

Three hepatologists and one radiologist independently assessed the results to obtain the LI-RADS score (DCY with 20 years of clinical experience, YJC with 13 years of clinical experience, and JP with 7 years of clinical experience). The final assessment was made by professor XLM (with 21 years of clinical experience).

A tumor is defined as having an LI-RADS score of 5 (LR-5) when presenting with a focal liver lesion (FLL) (>10mm) showing arterial phase hyperenhancement (APHE) and nonperipheral “washout” (WO) regardless of the presence of an enhancing capsule (EC). A tumor is defined as LR-4 if any of the following apply: (a) it is >20mm in size and shows APHE without WO or EC, or else shows no APHE with EC; (b) it is 10-19mm in size and shows APHE with EC and no WO; (c) it is <10mm in size and shows APHE with WO or EC. A tumor is defined as LI-3 if any of the following apply: (a) it is <20mm in size and shows APHE (no rim) with no WO or EC; (b) it shows no APHE and no EC; (c) it is <20mm in size and shows no APHE and has EC. Given that all patients studied were diagnosed with HCC, we did not use the category LI-RADS-M, which indicates cases as either ‘probably’ or ‘definitely’ malignant, non-specific to HCC.

### Histopathological evaluation

After being fixed in 4% paraformaldehyde (pH 7.4, for 24 hours), resected samples were dehydrated, and embedded in paraffin. The nesting tissues were sectioned continuously at a thickness of 3.0 μm. The tissue slides were xylenated, hydrated in a series of alcohols, and then submerged in phosphate-buffered saline (PBS). Antigen retrieval was achieved in ethylene diamine tetraacetie acid (EDTA) buffer (pH 8.0, 100°C, 2.5 minutes). After washing with PBS, 100 μL of endogenous peroxidase blocking agent (3% H2O2) was added and incubated for 10 minutes at room temperature. Slides were incubated with primary antibodies against human VEGFR2 (1:500, ZSGB-BIO, Beijing, China, ZA-0287) for 60 minutes at 37°C. Next, they were treated with enzyme-labelled goat anti-mouse immunoglobulin G (IgG) polymer (ZSGB-BIO, Beijing, China, IB000087) for 30 minutes at 37°C. Then, 100 μl diaminobenzidine reagent (ZSGB-BIO, Beijing, China, MA-2000) was added and incubated at room temperature for 6 minutes. After washing with tap water, stained with hematoxylin staining solution for 30-60 seconds at room temperature, differentiate with hydrochloric acid and alcohol, and return to blue. All experiments were performed by the same team. The operators were independent of the diagnostic results.

We utilized the Remmele/Stegner immunoreactive score for the semi-quantitative assessment of VEGFR2 staining (IRS score) (19). All results were evaluated independently by YY (with 15 years of clinical experience) and JC (with 20 years of clinical experience). The extent scores representing the intensity of staining were “0” (unstained, negative), “1” (brown stained, weakly positive), “2” (dark brown stained, moderate positive), or “3” (darker brown, strongly positive). IRS scores were calculated by multiplying the extent scores with a factor based on the percentage of tumor cells that were positive (0–10%/1; 10–50%/2; 50–80%/3; 80–100%/4). We defined the IRS scores 0, 1, 2, 3 as negative and 4, 6, 8, 9, 12 as positive. For the cases where the pathological diagnosis results and histological staining results were not uniform, we adopted the feature that occupied a large area as the judgment result of the tumor.

### Statistical analysis

For continuous variables, the Mann-Whitney U test was used. For categorical variables, the Chi-square test or Fisher’s test was used where necessary. Univariate analysis was used to screen for potentially significant variables. Variables with p<0.05 that were screened out from the univariate analysis were entered into the multivariate analysis to determine the independent predictors. The “rms” package in R 3.5.2 (https://www.r-project.org/) was used to generate a nomogram utilizing the significant predictors. The internal discrimination validation was performed using the “pROC” package and 1,000 iterations of bootstrapping. We derived the corresponding AUC value, sensitivity, and differentiation specificity with 95% confidence intervals. Using the calibration plot of the ‘rms’ package, we visually evaluated the degree of either over- or underestimating the expected probability compared to the actual VEGFR2-positivity, which was internally verified with 1,000 cycles of bootstrapping. As previously stated, the “rmda” package was used to conduct decision curve analysis (DCA). SPSS 25.0 (IBM, USA) was used to conduct the statistical analysis. The statistical significance was set at p<0.05 for all two-sided tests.

## Results

**Figure 2:**
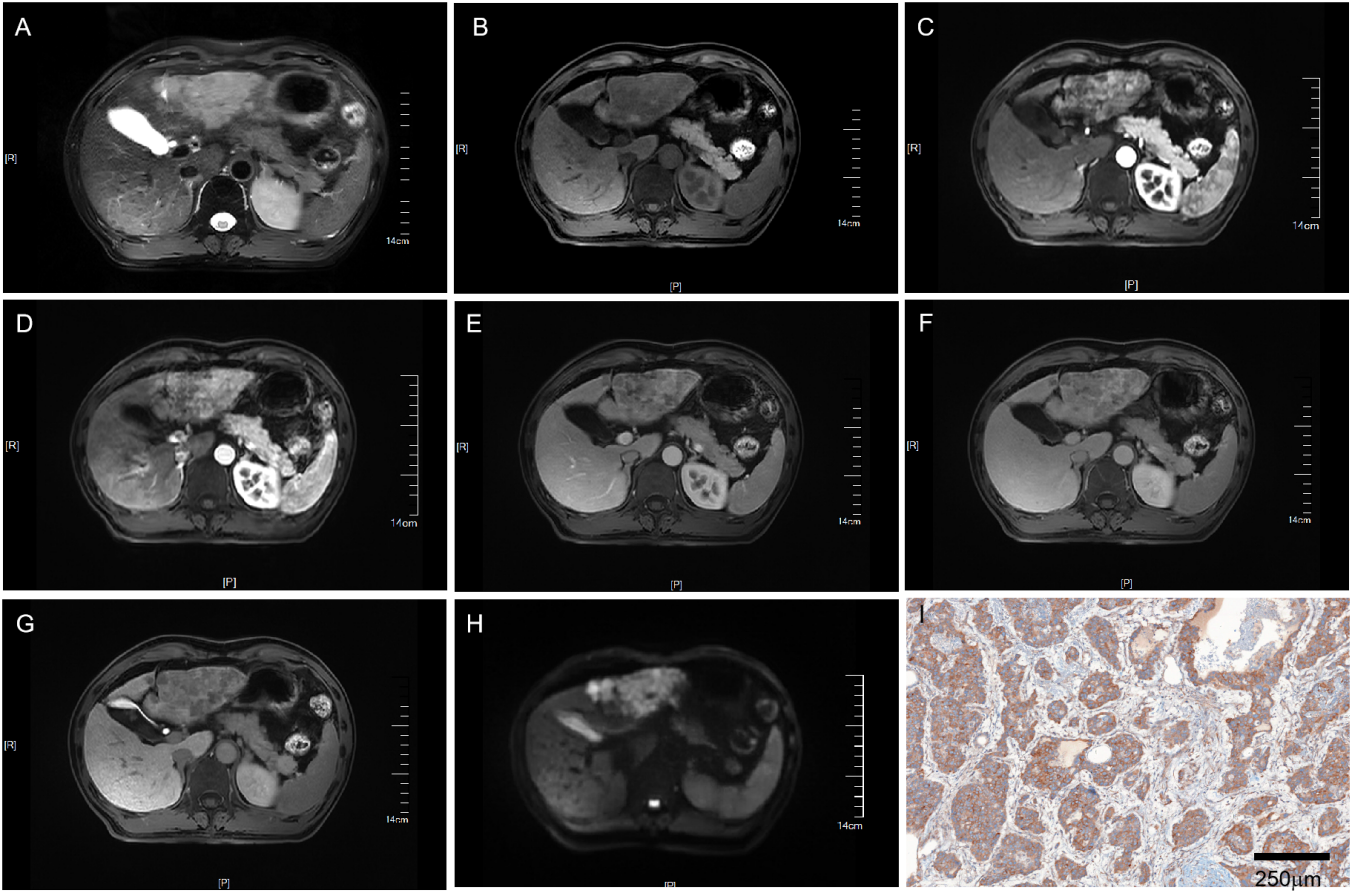
MRI of a VEGFR2-positive HCC patient with LI-RADS 5. Patient summary: a 50-year-old man with HCC in the left lobe of the liver, AFP level of 266ng/ml, lesion dimension on MRI of 8.1cm, and LI-RADS score of 5. **(A)** Axial T2-weighted MRI, **(B)** Axial T1-weighted MRI. **(C, D)** Early (15s) and late arterial phase (25s). **(E)** Portal venous phase (1min). **(F)** Delay phase (3min). **(G)** Hepatobiliary phase (15min). **(H)** Axial diffusion-weighted MRI. **(I)** VEGFR2-positivity in pathology. MRI, magnetic resonance imaging; LI-RADS, liver imaging reporting and data system; AFP, alpha-fetoprotein; VEGFR2, vascular endothelial growth factor receptor 2.

**Figure 3:**
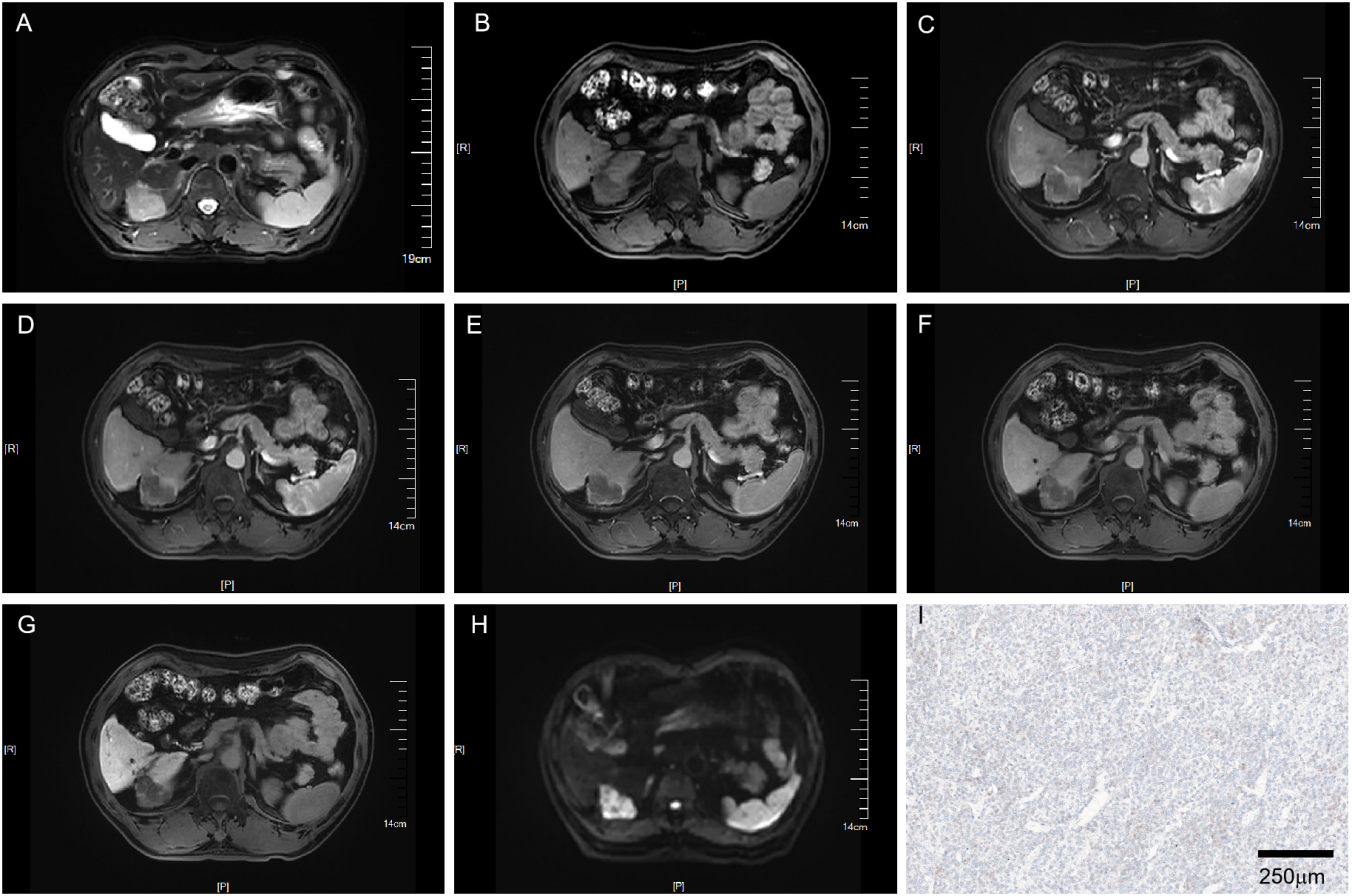
MRI of a VEGFR2-negative HCC patient with LI-RADS 3. Patient summary: a 59-year-old man with HCC in the right lobe of the liver, AFP level 2.7ng/ml, lesion dimension on MRI 3.8cm, and LI-RADS score of 3. **(A)** Axial T2-weighted MRI. **(B)** Axial T1-weighted MRI. **(C, D)** Early (15s) and late arterial phase (25s). **(E)** Portal venous phase (1min). **(F)** Delay phase (3min). **(G)** Hepatobiliary phase (15min). **(H)** Axial diffusion-weighted MRI. **(I)** VEGFR2-negative in pathology. MRI, magnetic resonance imaging; LI-RADS, liver imaging reporting and data system; AFP, alpha-fetoprotein; VEGFR2, vascular endothelial growth factor receptor 2.

**Table 1** provides a summary of the 206 patients’ features. Of the 206 patients, 160 had a single tumor and 46 had multifocal tumors. For patients with multifocal tumors, we took the tumor with the largest diameter for each patient for subsequent study. Following a thorough pathological examination, these individuals were classified as either VEGFR2-positive (86/206, 41.7%) or VEGFR2-negative (120/206, 58.3%) according to the immunohistochemical results. Patients positive for VEGFR2 were confirmed to have higher serum AFP levels, larger tumor dimensions on ceMRI, higher LI-RADS scores, and larger tumor dimensions on final pathology (all p<0.001). In univariate logistic regression model analysis, tumor dimensions on both the ceMRI and LI-RADS scores were shown to be closely linked with the high expression of VEGFR2 (**Table 2**). In multivariate logistic analysis, only two predictors remained significant: AFP (odds ratio 7.648, 95% confidence intervals (CI) 2.849-20.531 p<0.001) and the LI-RADS score (odds ratio 30.430, 95% CI 10.278-90.096, p<0.001) (**Table 3**). Representative cases illustrating positive and negative VEGFR2 expression in resected samples were shown in **Figure 2** and **3**, respectively.

**Table 1:** Characteristics of the 206 patients stratified by the VEGFR2 status of HCC.

Features	N=206	VEGFR2		p
					Negative (120)	Positive (86)	
Age (y)	59 (58-61)	60 (58-62)	58 (55-60)	0.126
Gender (male)	175 (85.0)	105 (87.5)	70 (81.4)	0.227
WBC (10^9/L)	5.4 (5.1-5.6)	5.3 (5.0-5.7)	5.4 (5.0-5.8)	0.956
Platelets (10^9/L)	153.4(143.3-163.2)	149.2 (136.7-161.7)	159.0 (142.4-175.5)	0.434
ALT (U/L)	54.2 (31.1-77.4)	68.7 (29.2-108.3)	34.0 (28.6-39.4)	0.353
AST (U/L)	64.8 (28.8-100.8)	85.9 (24.1-147.7)	35.4 (30.1-40.7)	0.698
TB (umol/L)	17.2 (13.3-21.0)	18.4 (11.9-25.0)	15.4 (13.6-17.2)	0.404
AFP (ng/ml)				<0.001
<400	164 (79.6)	107 (89.1)	57 (66.3)	
>400	42 (20.4)	13 (10.8)	29 (33.7)	
HBV infection				0.406
+	184 (89.3)	109 (90.8)	75 (87.2)	
-	22 (10.7)	11 (9.2)	11 (12.8)	
Liver cirrhosis (+)				<0.001
+	91 (44.2)	39 (32.5)	52 (60.5)	
-	115 (55.8)	81 (67.5)	34 (39.5)	
Tumor dimension on ceMRI (cm)	4.8 (4.3-5.2)	4.1 (3.6-4.7)	5.7 (4.9-6.4)	<0.001
LI-RADS score				<0.001
3	63 (30.6)	57 (47.5)	6 (7.0)	
4	53 (25.7)	36 (30)	17 (19.8)	
5	90 (43.7)	27 (22.5)	63 (73.2)	
Tumor dimension on pathology (cm)	4.8 (4.3-5.2)	4.1 (3.6-4.7)	5.7 (4.9-6.4)	<0.001
pT stage				0.467
T1	105 (51.0)	65 (54.2)	40 (46.5)	
T2	74 (36.0)	39 (32.5)	35 (40.7)	
T3-4	27 (13.0)	16 (13.3)	11 (12.8)	
Edmondson-Steiner grade				0.908
I-II	92 (44.7)	54 (45)	38 (44.2)	
III-IV	114 (55.3)	66 (55)	48 (55.8)	
Focality				0.343
single	160 (77.7)	96 (80)	64 (77.4)	
multiple	46 (22.3)	24 (20)	22 (25.6)	
MVI				0.669
Positive	59 (28.6)	33 (27.5)	26 (30.2)	
Negative	147 (71.4)	87 (72.5)	60 (69.8)	

Continuous variables are presented as media (interquartile range, IQR), while categorical variables are presented as patients (%).

WBC, white blood cells; ALT, alanine aminotransferase; AST, aspartate; aminotransferase; AFP, alpha-fetoprotein; HBV, hepatitis B virus; ceMRI, contrast-enhanced magnetic resonance imaging; LI-RADS, Liver Imaging Reporting and Data System; MVI, microvascular invasion.

**Table 2:** Univariate logistic regression analyses for the prediction of VEGFR2-positive HCC.

Variable and intercept	Univariate logistic regression	
		OR (95%CI)	p
Age	0.979 (0.955-1.005)	0.111
Gender (male vs. female)	0.625 (0.290-1.345)	0.229
WBC (10^9/L)	1.021 (0.891-1.170)	0.768
platelets (10^9/L)	1.002 (0.998-1.006)	0.344
ALT (U/L)	0.994 (0.986-1.002)	0.160
AST (U/L)	0.997 (0.989-1.004)	0.393
TB (μmol/L)	0.995 (0.982-1.008)	0.478
AFP (>400ng/ml)	4.188 (2.020-8.680)	<0.001
HBV (+)	0.688 (0.284-1.669)	0.408
Liver cirrhosis (+)	3.176 (1.784-5.656)	<0.001
Tumor dimension on ceMRI (cm)	1.163 (1.060-1.277)	0.001
LI-RADS (5 vs. 3/4)	22.167(8.535-57.570)	<0.001

CI, confidence interval; OR, odds ratio; WBC, white blood cells; ALT, alanine aminotransferase; AST, aspartate aminotransferase; AFP, alpha-fetoprotein; HBV, hepatitis B virus; ceMRI, contrast-enhanced magnetic resonance imaging; LI-RADS, Liver Imaging Reporting and Data System.

**Table 3:** Multivariate logistic regression analyses for the prediction of VEGFR2-positive HCC.

Variable and intercept	Multivariate logistic regression	
		OR (95%CI)	p
AFP (>400ng/ml)	7.648 (2.849-20.531)	<0.001
Tumor dimension on ceMRI (cm)	1.036 (0.921-1.164)	0.558
LI-RADS (5 vs. 3/4)	30.430(10.278-90.096)	<0.001

AFP, alpha-fetoprotein; ceMRI, contrast-enhanced magnetic resonance imaging; LI-RADS, Liver Imaging Reporting and Data System.

Furthermore, we tested the diagnostic performance of AFP and the LI-RADS score for VEGFR2-positivity in HCC using final pathology as a reference. The AUC area, sensitivity, and specificity of the LI-RADS scores were 0.790, 0.733, and 0.775, respectively. The AUC area, sensitivity, and specificity of AFP were 0.614, 0.337, and 0.892, respectively (**Supplementary Figure 1**).

**Figure 4:**
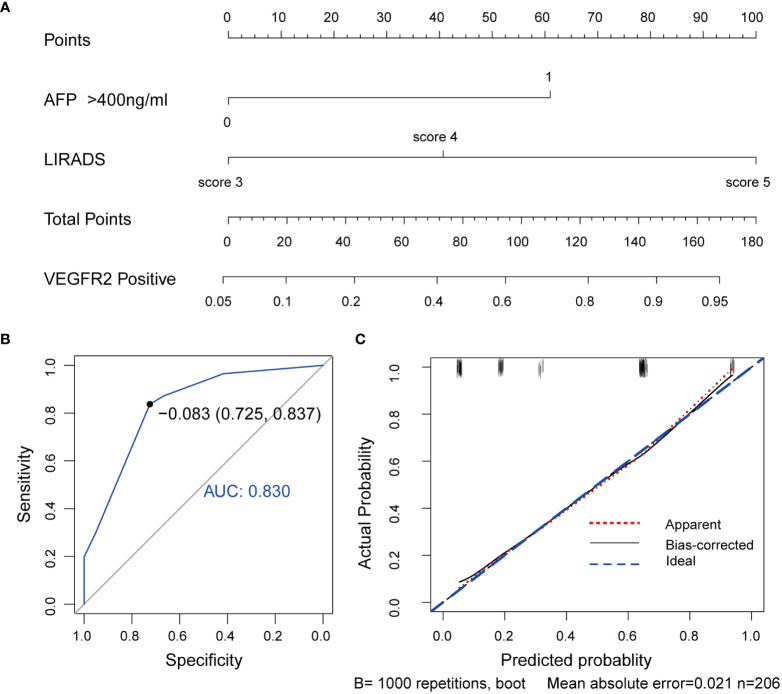
VEGFR2-positive-risk nomogram establishment and validation. **(A)** Risk nomogram incorporating AFP and the LI-RADS score for predicting high VEGFR2 expression in HCC patients. **(B)** ROC curve for the nomogram’s VEGFR2-positive prediction AUC, sensitivity, and specificity. **(C)** Calibration curves illustrating the nomogram’s calibration in terms of the agreement between the predicted risk of high VEGFR2 expression and the actual pathological VEGFR2 expression status. The 45˚blue line represents a perfect prediction, the red dashed line shows the nomogram’s predictive performance, and the black solid line is bias-corrected. The nomogram’s forecast accuracy improves as the dashed line approaches the ideal line. AUC, area under the curve; AFP, alpha-fetoprotein, LI-RADS, Liver Imaging Reporting and Data System, VEGFR2, vascular endothelial growth factor receptor 2, ROC, receiver operating characteristic.

**Figure 5:**
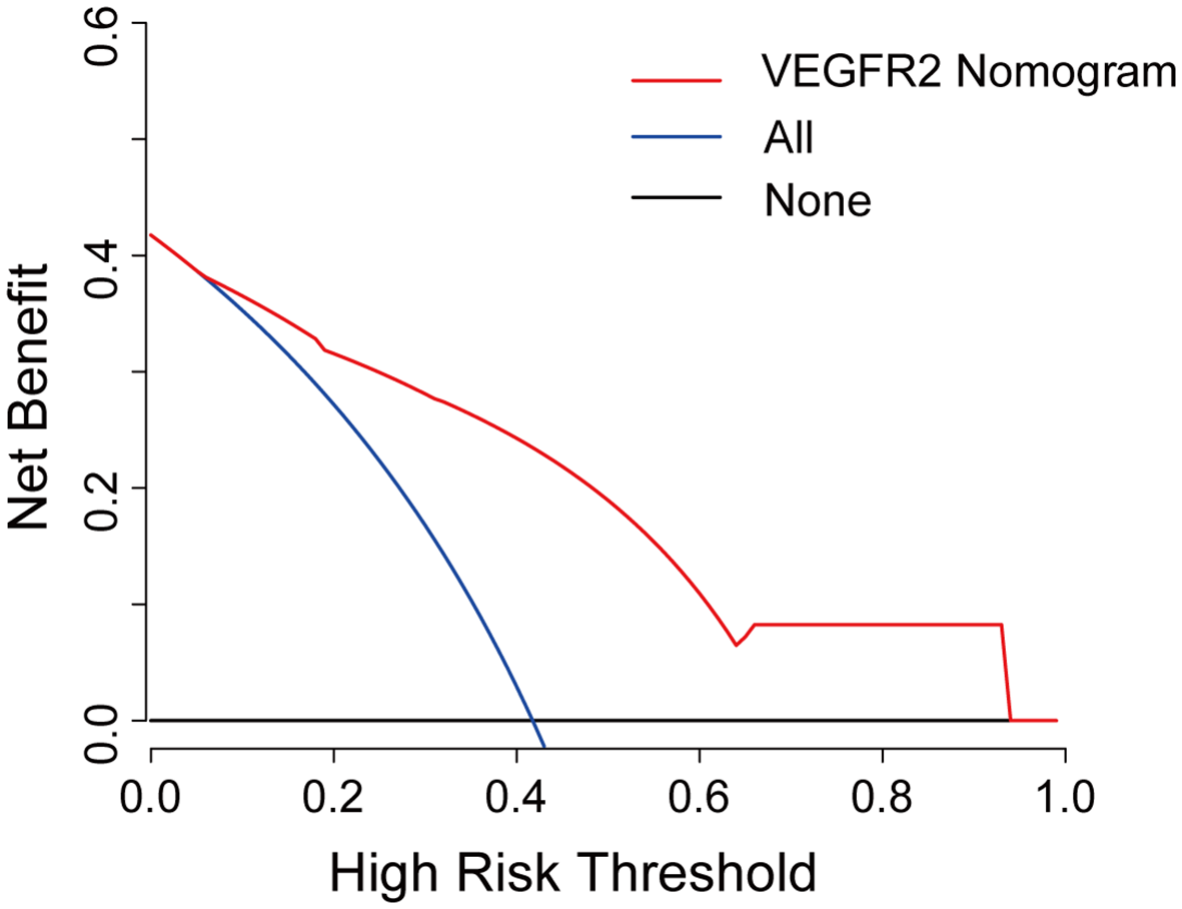
Decision curve analysis for the VEGFR2-positive-risk nomogram. The red line represents the nomogram, the blue line represents the condition that all patients are VEGFR2-positive, and the black line represents the condition that no patient harbors detectable VEGFR2 expression. The decision curve reveals that utilizing the VEGFR2-positive-risk model to forecast VEGFR2 expression offers a greater benefit than intervening in either all or none of the patients when the threshold probability ranges from 0.07 to 0.95.

Further, a VEGFR2-positive risk nomogram integrating these two predictors was created (**Figure 4A**). Bootstrapping was used to verify the nomogram internally. Nomogram discrimination was performed by ROC analysis with an AUC of 0.830 (95% CI 0.750, 0.866) (**Figure 4B**). The sensitivity and specificity of the results were 0.837 and 0.725, respectively. Furthermore, with a mean absolute error of 0.021, a bootstrapped calibration curve of the model revealed no adverse departure of the expected risk from the actual risk of VEGFR2-positivity (**Figure 4C**). The DCA curve was used to investigate its clinical benefits (**Figure 5**). With threshold probabilities ranging between 0.07 and 0.95, the model provides larger net advantages than intervening in either all or none of the patients.

## Discussion

As far as we know, this is the first research of its kind in developing a model to predict VEGFR2 expression in HCC using a combination of clinical features and MRI data. The related sensitivity and specificity of the nomogram were 0.725 and 0.837, respectively.

VEGFR2, which is abundantly expressed in HCC and vessels, is linked to tumor biological behavior and 5-year survival rate in HCC patients (20). In our study, the disease-free survival (DFS, p=0.003) and overall survival (OS, p=0.010) time of the groups stratified by AFP (400 ng/ml) were significantly different (**Supplementary Figure 2B, D**). However, the DFS and OS of the subgroups divided by LI-RADS score and VEGFR2-positivity were not significantly different, possibly due to the sample size. Postoperative adjuvant therapy was also one of the critical factors influencing OS and DFS. VEGFR2 is a crucial target in anti-cancer therapy because it is an essential regulator of angiogenesis (21). Chu et al. revealed high VEGFR2 expression to be correlated with hepatic cirrhosis, and this finding was consistent with the results of the current study (17). However, HBV infection was not found to be a predictor of high VEGFR2 expression, and this result may have been partly due to differences in the conditions and nationality of the patients in our center. In our study, the AFP level (>400 ng/ml), presence of liver cirrhosis (confirmed by pathology), and LI-RADS score were revealed to be independent predictors of VEGFR2 expression in HCC patients. Given that the LI-RADS scoring system is suitable for patients with either HBV infection, liver cirrhosis, or a history of HCC, we did not include liver cirrhosis in the model in order to minimize selection bias (13).

Previous research has shown that the LI-RADS score is associated with MVI, postoperative disease-free survival, and overall survival in HCC patients (22–25). Tumor proliferation and invasion require ample blood supply, and VEGFR2 is regarded to be one of the most essential regulators of angiogenesis (21). We speculated that the LI-RADS score could reflect the blood supply of hepatocellular carcinoma to a certain extent, and therefore illustrate the expression of VEGFR2 in HCC. Indeed, we found that the LI-RADS score was strongly correlated to the expression of VEGFR2 (odds ratio 40.825, p<0.001). Future studies using additional research centers and larger samples can further validate this finding. AFP has traditionally served as a biomarker for HCC, and its relationship with the behavior and prognosis of HCC has been validated (26, 27). We further validated the strong correlation between AFP and VEGFR2 expression, and these findings were consistent with the findings of Huang J et al. (20). It is possible that AFP is secreted more as the tumor proliferates, as VEGFR2 expression is positively correlated with tumor size (28). To explain its correlation from another perspective, high levels of serum AFP and the high expression of VEGFR2 both have been shown to predict poorer prognosis (20, 29), but the causal relationship remains unclear, and more studies are needed to further explore this.

Due to the poor performance of various factors in predicting VEGFR2 expression, the construction of additional models will be necessary. Based on recent studies that have observed significant differences in clinical and MRI parameters between high and low VEGFR2 expression, we aimed to establish a risk model that could predict high VEGFR2 expression. Variables incorporating both AFP levels and LI-RADS scores were found for nomogram generation using multivariate logistic regression analysis. With internal validation, the VEGFR2-positive-risk nomogram revealed a high degree of discrimination and calibration as well as significant net advantages under particular threshold probabilities. For individuals diagnosed with HCC, the associated VEGFR2-positivity risk may be computed using this nomogram. The risk of VEGFR2-positivity based on the nomogram suggests that this population might be more suitable for targeted therapies involving appropriately targeted drugs. This would be beneficial for the individualized treatment of HCC patients, as well as advantageous in reducing both medical costs and the social burden.

Our research has some limitations. Firstly, because the study had a retrospective design, there will certainly have been some degree of selection bias Secondly, based on the postoperative pathological results of all our patients being consistent with HCC, we did not use the classification category LI-RADS-M, and so the conclusions may be biased. Furthermore, this study only found a correlation between the LI-RADS score and VEGFR2 expression without offering a convincible mechanistic cause. We therefore advise that further studies be performed for verification and elucidation. Thirdly, in cases involving multiple lesions, we only selected the largest lesions for study, which introduced unreliability due to tumor heterogeneity. Fourthly, we only studied the ability of MR to discriminate VEGFR2. Other necessary imaging tests, such as contrast-enhanced CT and ultrasonography, are warranted to provide further data. Lastly, this was a unicentric respective study. We only conducted internal validation because of the sample size, and hence, external validation sourced from other centers will be required in the future.

## Conclusion

In conclusion, both the AFP level and LI-RADS score are significant predictors for VEGFR2 expression in HCC. By integrating them, we built a model to predict VEGFR2 expression among HCC patients. With internal validation, the model demonstrated good diagnostic performance. Considerable net advantages could be acquired under particular threshold probabilities.

## Data availability statement

The raw data supporting the conclusions of this article will be made available by the authors, without undue reservation.

## Ethics statement

The studies involving human participants were reviewed and approved by Nanjing Drum Tower Hospital Medical Ethics Committee. The patients/participants provided their written informed consent to participate in this study. Written informed consent was obtained from the individual(s) for the publication of any potentially identifiable images or data included in this article.

## Author contributions

LZ and CC contributed equally. DY and XM are the corresponding authors. All authors contributed to the article and approved the submitted version.

## Funding

This work was supported by the National Natural Science Foundation of China (No. 81871967).

## Conflict of interest

The authors declare that the research was conducted in the absence of any commercial or financial relationships that could be construed as a potential conflict of interest.

## Publisher’s note

All claims expressed in this article are solely those of the authors and do not necessarily represent those of their affiliated organizations, or those of the publisher, the editors and the reviewers. Any product that may be evaluated in this article, or claim that may be made by its manufacturer, is not guaranteed or endorsed by the publisher.
