# Supplementary figures and images for: Combined clinical features and MRI parameters for the prediction of VEGFR2 in hepatocellular carcinoma patients
Source: Front Oncol. 2022 Oct 13;12:961530. doi: 10.3389/fonc.2022.961530 (PMC9608502; doi:10.3389/fonc.2022.961530)

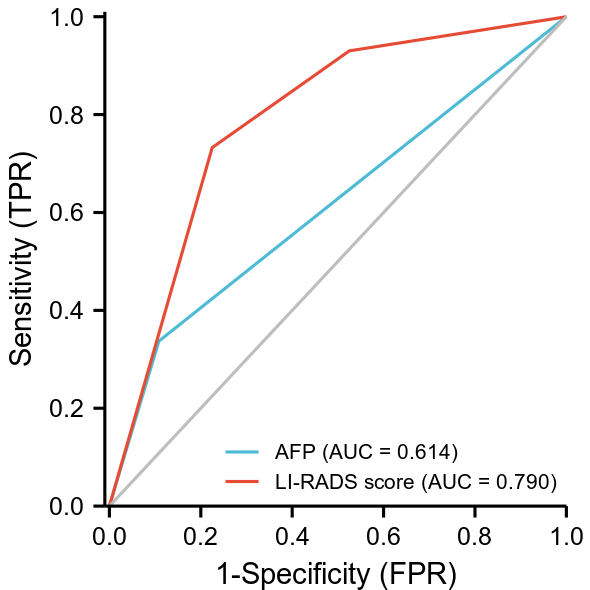
Supplementary Figure 1

Supplementary Figure 2


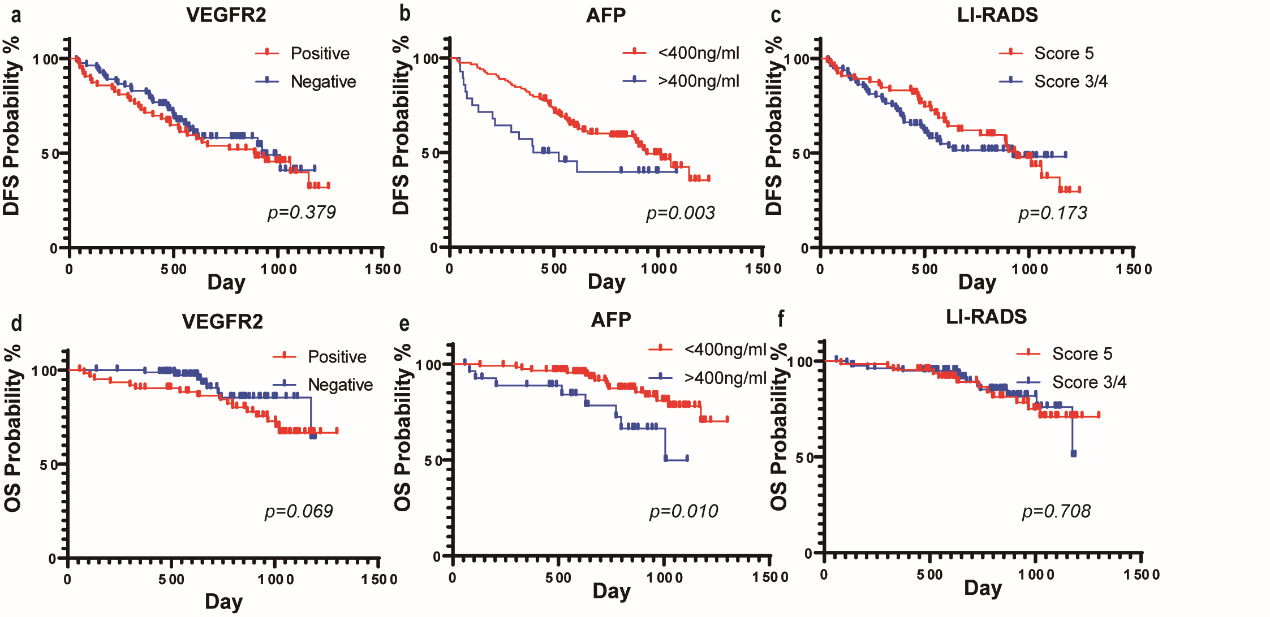

Supplement: Supplementary file 1 [file DataSheet_1.docx]
